# Perspectives in translating microfluidic devices from laboratory prototyping into scale-up production

**DOI:** 10.1063/5.0079045

**Published:** 2022-03-17

**Authors:** Hengji Cong, Nan Zhang

**Affiliations:** 1 Centre of Micro/Nano Manufacturing Technology (MNMT-Dublin), School of Mechanical & Materials Engineering, University College Dublin, Dublin 4, Ireland; 2 MiNAN Technologies, Dublin 4, Ireland

## Abstract

Transforming lab research into a sustainable business is becoming a trend in the microfluidic field. However, there are various challenges during the translation process due to the gaps between academia and industry, especially from laboratory prototyping to industrial scale-up production, which is critical for potential commercialization. In this Perspective, based on our experience in collaboration with stakeholders, e.g., biologists, microfluidic engineers, diagnostic specialists, and manufacturers, we aim to share our understanding of the manufacturing process chain of microfluidic cartridge from concept development and laboratory prototyping to scale-up production, where the scale-up production of commercial microfluidic cartridges is highlighted. Four suggestions from the aspect of cartridge design for manufacturing, professional involvement, material selection, and standardization are provided in order to help scientists from the laboratory to bring their innovations into pre-clinical, clinical, and mass production and improve the manufacturability of laboratory prototypes toward commercialization.

## I. INTRODUCTION

Innovative and qualified products in life science always come with cross-functional research. Microfluidic devices, as a typical example, rise from the collaboration of interdisciplinary fields, including biology, chemistry, medicine, fluid dynamics, material science, optics, and electronics. In general, microfluidics refers to the manipulation or processing of liquids in a small volume (10^−6^–10^−9^ l) within microminiaturized chambers or channels. In such a case, a sample reaction and detection can be integrated into a small microfluidics platform. When the scaling of processes in a laboratory is down to a chip-based platform, the system is generally regarded as “lab-on-a-chip” (LOC). There are plenty of other similar systems according to various operation formats like lab-in-a-tube,^1^ lab-on-a-disk (LOAD),^2^ and microfluidic paper-based analytical devices (microPADs).^3^ Other microstructured components in which a body fluid or solution will flow when they are used can also be defined as microfluidic devices, such as a liquid dispensing tool and a flow control component. Among them, microfluidic chips dominate the microfluidic device market with 85% market share in value.^4^

According to Yole Development, the microfluidic product market was valued around U.S. $11 billion in 2019 and will reach U.S. $24.5 billion in 2025, with a compound annual growth rate (CAGR) of 14%.^5^ Based on application scenarios, microfluidic devices can be mainly utilized for environmental surveillance,^6^ food safety analysis,^7^ pharmaceutical research,^8^ clinical and veterinary diagnosis,^9^ drug delivery,^10^ and point-of-care testing (POCT).^11,12^ Areas like optical imaging,^13^ forensic residue analysis,^14^ and space science^15^ also build collaborations with microfluidic techniques. Among these applications, point-of-care testing keeps the largest share of the market.^16^ In 2003, the WHO published a set of guidelines to describe the standard characteristics of a diagnostic test referred to as “ASSURED,” which can be summarized as affordable, sensitive, specific, user-friendly, rapid, equipment-free, and deliverable to end-users.^17^ Microfluidics-based POCT devices are highly compatible with these criteria, laying the foundation for its market development. The SARS-CoV-2 outbreak, to a great extent, also accelerates the developing speed of POCT.^18^ Our World in Data (OWID) reported in the United States, around 209 × 10^6^ COVID-19 tests were conducted in the first six months of 2021,^19^ not to mention the whole world.

**FIG. 1.**
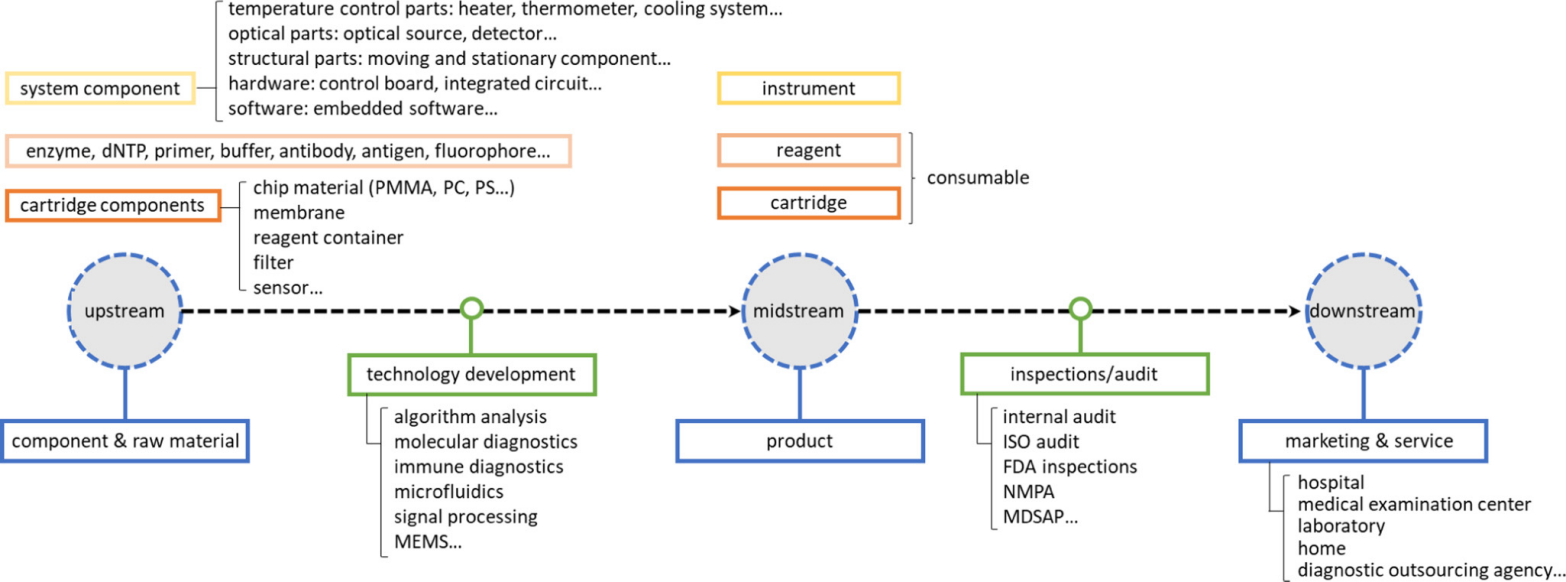
Industrial supply chain of microfluidic devices.

Based on the product type, the global microfluidic device market can usually be segmented into instrument, software, reagent, cartridge, and others. Consumables including reagents and cartridges contribute a dominating share to the market. To have a better insight into how a microfluidic device is delivered to end-users, the industrial supply chain of microfluidic devices is given in a brief manner, as shown in Fig. 1. The industrial supply chain of microfluidic products is divided into (1) upstream: components and raw materials, (2) midstream: products including instrument and consumables (reagent and cartridge), and (3) downstream: marketing and services delivered to end-users. To generate a successful product, various techniques are under development to ensure that each part inside the machine is working properly. Also, as the majority of microfluidic devices market goes to POCT products,^16,20^ a systematic product audit is necessary to evaluate if the device is produced in compliance with regulatory requirements before it enters the market. This process, including regular internal audits and external audits (ISO, FDA, NMPA, etc.), determines whether the device is safe and qualified through an efficient assessment. The Medical Device Single Audit Program (MDSAP) allows companies to contact with multiple countries through a single regulatory quality management system and the FDA will accept it as a substitute for FDA inspections.^21^

Although the industrial supply chain is currently expanding and abundant research articles have been published in various peer-reviewed journals, the diffusion of microfluidics from research into customer products has not yet lived up to its early advertising.^22^ Such an unexpected outcome is attributed to several reasons.^23^ First, current microfluidic applications are mostly in the development stage or early stage of their product life cycle, and there remains a gap between the technologies and the end-user markets.^24^ To find a “killer application” is perceived as one approach to trigger widespread adoption of microfluidics.^24,25^ However, to define the niche market for a specific microfluidic device requires much effort on understanding customer requirements and application scenarios and benchmarking with existing solutions. Second, the fundamental research carried out by academic researchers is random and unfocused, while commercialization requires a better understanding of market needs and bottlenecks.^26^ Enhancing interdisciplinary communication is required to keep the team aligned and every effort focused on a specific problem. Third, the fundraising process is lengthy and complex. The development of a microfluidic product requires high investment due to its multidisciplinary nature.^24^ Not only the clinical market but also investor sentiment needs to be thoroughly investigated^12^ for a stable and continuous investment from the seed stage. This process is tedious and tough for both investors and entrepreneurs. Finally, mass production of microfluidic devices can be effortful in terms of scalability and cost-effectiveness. Other barriers, such as the comprehensive process of regulatory approval^27^ and patent licensing^16^ for clinic diagnostic products, may also slow down the pace of commercialization.

Among the barriers listed above, mass production is a challenging task and is also a fundamental factor to determine whether a design concept from the laboratory can be eventually translated into a product. Particularly for microfluidics, when its applications remain in areas like molecular diagnostics, there are many competitive products, such as integrated microfluidic platforms for Polymerase Chain Reaction (PCR) testing. Scaling up the manufacturing of disposable microfluidic cartridges with lower cost and shorter cycle time will hugely enhance the competitiveness of the microfluidic solution with its counterparts.

In this Perspective, we will focus on the mass production of microfluidic devices. We will first share our viewpoints on the manufacturing challenges to confront when translating microfluidic devices from laboratory research to industry products (Sec. II). Then, the concept development is briefly introduced as it is an essential step before the subsequent manufacturing phase (Sec. III). By disassembling the manufacturing process chain into detailed fabrication steps, we aim to provide an explicit roadmap of the translation process with the focus on scale-up technologies (Sec. IV). Standardization is also brought up for its significant role in accelerating such a translation process (Sec. V). Finally, we will share some of our views for tackling the translation challenges (Sec. VI).

## II. CHALLENGES IN SCALE-UP FABRICATION

Microfluidics has evolved rapidly from the concept of “miniaturized Total Analysis System” (μTAS) into the emerging field with enormous commercial values over the past 30 years.^28^ The fundamental research of microfluidics matures this technology, making it ready for commercial exploitation. Funding agencies also show a higher interest in projects with a higher translation probability or co-production of research with industry to obtain a tangible return on their investments.^23^ This commercialization culture in academia drives researchers to bring microfluidic solutions from lab to market to tackle real-world problems,^29^ such as the rapid diagnosis of SARS-CoV-2 virus.^30^ Meanwhile, plenty of academic spin-offs have been established based on the innovative character of their technology, such as Stilla Technologies from the laboratory of hydrodynamics (LadHyX) at Ecole Polytechnique,^31^ Fluid-Screen from Yale University,^32^ and BluSense Diagnostics from Technical University of Denmark.^33^ These activities have confirmed the increasing popularity of academic entrepreneurship among higher education institutions worldwide.^34^

As discussed in Sec. I, unlike early-stage discovery of microfluidics where more researchers mainly focused on the fundamentals understanding of fluidic design principles and operation of biological particles, and other inventions, more and more researchers are developing a real-world useful device toward commercialization to meet specific market needs.^23^ In this sense, manufacturing techniques can differ hugely comparing the prototyping stage in academia and high-volume production in industry. Plenty of challenges will show up during the roadmap toward commercialization. A typical microfluidic system is mainly composed of a microfluidic cartridge, reagents, and an instrument. Here, we would like to highlight the challenges to translate laboratory prototyping into mass production of the microfluidic cartridge, which is the core of a microfluidic system.

**FIG. 2.**
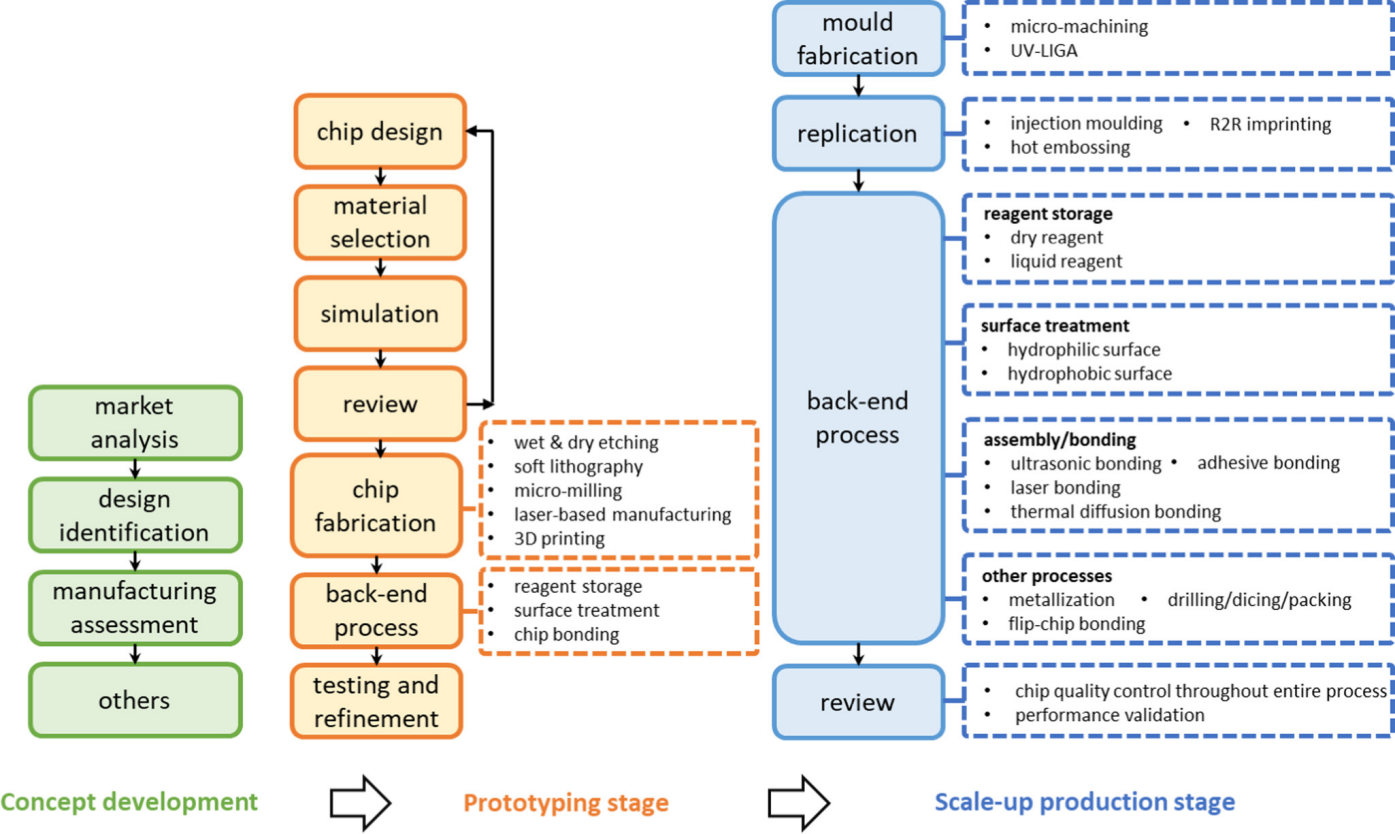
Development process of microfluidic devices in prototyping and scale-up production stages.

(1) Complexity of cartridge integration:^23^ Commercial microfluidic cartridges are integrated with multiple reaction chambers, biosensors, and microchannels for micro/nanoliter liquid processing, which has a high requirement for manufacturing accuracy and requires various micromanufacturing technologies. Materials needed for different components like connectors, valves, membranes, or sensors could vary a lot; thus, multimaterial manufacturing and multimaterial heterogeneous integration are required for process development. Moreover, assembly of these components will enhance the difficulties, especially when dedicate dry/wet reagents are handled during the process.
(2) Long development period:^35^ The development of microfluidic chips covers design and laboratory prototyping, pre-clinical validation, clinical validation, and mass production. In the prototyping period, a low quantity of microfluidic chips (5–50 chips) is required, which requires the manufacturing process to be flexible with less effort and fast enough to validate the design. When translated to the pre-clinical and clinical study, the quantity is increased to 100–1000 chips, where the chips have to be demonstrated with scale-up capability, and the plastic materials are generally required due to merits like low cost, biocompatibility, and excellent optical properties.^36,37^ The design of the chip should be as close as the production part to ensure consistency. For mass production, the microfluidic chip is further developed to be a microfluidic cartridge adaptable to the microfluidic instrument. Low-cost scale-up production is becoming important. More than 10 000 parts are required, where the production cycle should be short, and high quality and consistency are necessary. When the quantity is increased to more than 20 000 parts, automation may be required to achieve high consistency and lower cost. Such a process can take 3–5 years along with the reagent and instrument development. With additional time for system optimization and audit/inspection, a considerably long period is needed for a complete microfluidic system. Considering such a long development process, a strategic plan should be made for cartridge manufacturing.
(3) Complex manufacturing process^38^ and high failure rate: Microfluidic cartridge development will experience prototyping and scale-up production. There are many different techniques involved in different processes and process steps, as detailed in Fig. 2. The selection of a proper prototyping process should also ensure it is scalable with less effort for scale-up redevelopment, good material compatibility, and satisfactory manufacturing precision. Additionally, as microfluidic chip fabrication involves many micromanufacturing processes and requires the integration of multiple materials,^39^ the failure rate could be high. This is particularly true for processes that are not commonly applied for mass production, such as chip bonding, surface treatment, metallization, reagent integration, etc., where no standard protocols are available on the market such as injection molding. This could lead to an increased failure rate of the production process. Thus, the quality control and process optimization could take much effort.
(4) High cost:^40^ Because of the complex manufacturing process, high failure rate, and relatively low production volume at the early stage, the manufacturing cost of microfluidic devices could be high. For POCT tests, when the cost of reagents is relatively fixed, the price of a cartridge can determine the overall cost per test and eventually the competitiveness of the POCT product.
(5) Lack of standardization:^35,39,41^ A well-established standardization system will bring benefits in reducing the development time and manufacturing costs. Once the consensus standards have been developed on topics ranging from interface dimensions to testing methods, more resources and effort could be focused on other aspects like innovative chip designing. This would accelerate the development from both chip and system levels, and regulatory approvals.

In addition to the challenges mentioned above, other issues like the lack of professionals with cross-disciplinary skills, unsatisfactory results due to adapting the protocols from prototyping to mass manufacturing, and difficulties in maintaining the long-term performance of the cartridge (especially reagent performance) may occur as well. It is also important to mention that due to the long development period and multiple skill requirements in micromanufacturing, a large manufacturing company has less interest, particularly in low quantity production, to help researchers translate their design to the stage of pre-clinical and clinical studies. Therefore, a better understanding of manufacturing process chains of a microfluidic chip for researchers who are motivated by future commercialization is important to avoid the potential risk of failure in cartridge development.

## III. CONCEPT DEVELOPMENT

Before device manufacturing, a product concept is always needed for an approximate description of product format, workflow inside, and technologies involved. For microfluidic devices, the concept development phase can be generally considered from the following aspects:^42^

(1) Marketing analysis: articulate market opportunity for the device; identify lead users and collect customer needs; analyze competitive products (benchmarking).
(2) Design identification: generate a schematic of the device; assign the elements of the schematic into chunks; create a geometric layout; identify the fundamental and incidental interactions.
(3) Manufacturing assessment: list fabrication techniques required for the whole device; set supply chain strategy; estimate manufacturing cost and time; assess feasibility and scalability.
(4) Others: allocate project resources; investigate patents, literature studies, and regulations.

Manufacturing cost is a key determinant of the successful translation from a laboratory platform into a product. It usually depends on the profit margin per product and the quantity a firm can sell. Design for manufacturing (DFM) is a common methodology used in the concept development for lowering down manufacturing costs without sacrificing product quality. The DFM principle should also be applied to microfluidic device development. Five basic elements need to be considered: process, design, materials, environment, and compliance/testing. We will discuss the details on design specifications and materials in prototyping and scale-up production stages for microfluidic chips in Sec. IV. Since the microfluidic chips in mass production are mostly made of polymers, materials with lighter weight and less burden of recycling should be considered from the environmental aspect. Compliance and regulatory standards are of universal importance in microfluidics for their wide applications in medical devices.

Ideally, DFM should be involved in the early stage of the design process, well before the microstructure tooling has begun. It assures that the design concept can be manufactured while meeting quantitative key performance indicators (KPIs).^43^ In addition, properly executed DFM needs to include all the stakeholders: designers, engineers, contract manufacturers, mold builders, and material suppliers. This “cross-functional” DFM intends to assess the design at all levels, component, subsystem, system, and holistic levels, to ensure the design is optimized and does not have unnecessary cost embedded in it.

## IV. MANUFACTURING PROCESS CHAIN

Before the manufacturing of a commercialized cartridge, a prototyping period using polydimethylsiloxane (PDMS) based microfluidic chips usually comes at the first stage, especially for a product generated from academia. This period is mainly to test the methodology or basic functions of the chip, which is more related to the general design of chip structures or reagents. If the basic workflow for reactions on the chip is reasonable and realizable, a thermoplastic-based microfluidic chip is created in a low quantity. However, the material differences between PDMS and thermoplastics need to be noticed. Redevelopment may be required because of differences in surface properties, gas permeability, and moldability. Some techniques involved in the manufacturing like surface treatment or chip bonding may need to be optimized to suit thermoplastics. When the scheme for a thermoplastic chip is finalized, an integrated cartridge with the thermoplastic chip as the main body will come out after the addition of other supplementary components, as well as the pre-loaded reagents for sample reactions. In most cases, industry development skips the period with PDMS chip development, starting with thermoplastic chips in the prototyping period directly.

Microfluidic cartridge works as a hardware interface between the end-user and the instrument. For industry usage, the cartridge design depends on critical factors like cost, feasibility for volume manufacturing, assembly techniques, and ease of use. Typical examples include the BioFire Filmarray pouch with reagent reservoirs and a chemical circuit board in a vertical profile,^44^ the cobas Liat System assay tube with reagent package in different segments and separated by peelable seals,^45^ and the GeneXpert kit box with processing chambers, a PCR tube, and a valve body.^46^ Supplementary components in the cartridge are integrated to support liquid handling and signal detection. These components include membrane filters, sensor chips, valves, magnetic stirs, needles, etc.

The manufacturing process differs significantly from a PDMS chip in academia to a plastic cartridge in industry. Academic research is mainly for concept validation, while a commercialized product needs large-scale manufacturing with high repeatability. In such cases, the turnaround time for a piece of laboratory work should be short so that the design could be modified frequently during the concept validation process. Moreover, funding in academia is relatively lower compared with commercial investment. Low microstructured tooling cost is, therefore, preferred for research projects. For the industry, the cost for microstructured tooling fabrication can be compensated as the number of cartridges increases immensely.

Despite these differences, plenty of technologies are involved in both prototyping and scale-up production to complete a full manufacturing process chain. Figure 2 demonstrates the common workflow for the development of a microfluidic device, from concept development to prototyping and finally to scale-up production period. We herein attribute academic work and early-stage industrial work to the prototyping period, with the device quantity ranging from tens to hundreds. For the mass manufacturing period, the number of devices fabricated will reach 10 000 or even higher. The main processes in these two stages will be discussed in detail to illustrate the product roadmap.

### A. Prototyping stage (low volume: 10–1000 pieces)

#### 1. Chip specification

The specification of the microfluidic chip design is important, particularly when considering the next step for scale-up production. Here, we summarize the general chip specifications based on our manufacturing experience and some suggestions from Sony DADC:^47^

• Global flatness ≤300 *μ*m to ensure adequate interfacing with the optical, thermal, and pressurization systems of the instrument (microscope slide).
• High channel density while achieving local flatness ≤20 *μ*m/5 mm^2^.
• Very tight control (5%) over the channel width and height in a very narrow tolerance range over a total channel length of ∼100 mm.
• Feature size 5–500 *μ*m.
• Surface roughness <100 nm.
• Aspect ratio <1:1–1:2.

Such specifications are generally required for plastic microfluidic chips for potential scale-up. It could be a good reference for researchers in their initial design of microfluidic chips.

#### 2. Material selection

A variety of materials can be considered for the fabrication of microfluidic devices. Table I summarized the basic properties, fabrication methods, cost, and related products of some broadly used materials to facilitate direct comparison.

**TABLE I.** Comparison of materials used in microfluidic chips.^52,55^

Material		Silicon	Glass	Polymer					Paper
				PDMS	PS	PC	PMMA	COC/COP	
Property	Optical transparency	No	High	High	High	High	High	High	Low
	Thermal conductivity [W/(m k)]	High k = 149	Low k = 0.8	Low k = 0.2	Low k = 0.1–0.13	Low k = 0.19–0.22	Low k = 0.16–0	Low k = 0	Low
	Glass transition temperature (°C)	NA	NA	∼80^a^	92–100	145–148	100–122	70–155	NA
	Solvent resistance	High	High	Low	Low	High	High	Excellent	High
	Gas permeability	Low	Low	High	Low	Low	Low	Low	High
	Hydrophobicity	hydrophilic	hydrophilic	hydrophobic	hydrophobic	hydrophobic	hydrophobic	hydrophobic	Amphophilic
Fabrication methods		Wet etching, dry etching	Wet etching, reactive ion etching	Replica molding (mold mostly from soft lithography and CNC micromachining)	Injection molding, hot embossing	Hot embossing	Hot embossing, micromachining, laser ablation, injection molding	Injection molding	Photolithography, printing, cutting
Design freedom		Low	Low	High	High	High	High	High	Low
Mass manufacturing Capability		Low	Low	Low	High	High	High	High	High
Application		Digital PCR	Capillary electrophoresis, organic synthesis, droplet formation	Cell culture, organs on a chip	DNA synthesis, cell culture	PCR	DNA analysis, electrophoresis	Biochemical reactions, chip-HPLC^b^ application	Glucose detection, environment and food safety tests
Material cost		∼7$/4 in. wafer	0.15$/microscope slide (75 × 25 × 1 mm^3^)	∼150$/kg	<3$/kg	<3$/kg	2–4$/kg	11–35$/kg	NA

^a^ PDMS curing temperature.

^b^ HPLC: High Performance Liquid Chromatography.

**PDMS:** The most widely used material in academia is PDMS, which is a silicon-based organic polymer. Due to its biocompatibility and ease of fabrication, PDMS is popular for the transition from a design into quick tests in academia and, in some cases, as a precursor in industry. It should be noted that, besides the absorption of organic solvent and small molecules,^48^ other shortcomings like the high cost of production restrict PDMS to succeed in the commercial microfluidic arena. Becker^49^ also pointed out much of excellent academic work led to dead ends due to the failure in transferring results obtained with PDMS into other materials. It does not mean there is a lack of acceptance of PDMS in the commercial market. The point is, when choosing PDMS as the experimental material, one should consider more industrial aspects for further development. A full understanding of differences between PDMS and thermoplastic materials in both manufacturability and functionality is important to translate the design from PDMS to thermoplastic for design validation and scale-up.

**Silicon and glass:** Thanks to the growth of microelectromechanical systems (MEMS), silicon and glass dominate the fabrication of microfluidic devices as the first generation. For silicon, the manufacturing process is mature and properties like thermostability and chemical compatibility are favored.^38^ However, its opacity and high cost become the restrictions for commercial usage. Glass benefits from its optical transparency, ease of surface modification, and biocompatibility. But similar to silicon, glass-based devices suffer from the high manufacturing cost. Also, glass is fragile due to high rigidity, which results in the limitation of products based on glass substrates. Still, there are some popular products using silicon and glass-based platforms. For example, Quant Studio 3D Digital PCR system (Thermo Fisher Scientific) based on a silicon chip is used for profiling miRNAs in plasma samples.^50^ Patterned flow cell technology from Illumina takes the advantage of a glass substrate with patterned nanowells for diverse genomic applications.^51^ Compared to other materials, silicon and glass offer high stability, good precision, and manufacturing repeatability. It suits well for cases of harsh environment or high-precision applications.

**Thermoplastics:** Compared with the materials mentioned above, thermoplastics are more commonly used in industry. Thermoplastics can be processed when reaching glass transition temperature (T_g_) and retain their shapes after the cooling process. They are rigid, transparent, biocompatible, and capable of integration with electrodes. Materials including polystyrene (PS), polycarbonate (PC), polymethyl methacrylate (PMMA), and cyclic olefin copolymer (COC)/cyclic olefin polymers (COPs) are widely used as thermoplastics. These polymers also owe their success to the ease of mass production, reducing the complexity and fabrication cost in commercial applications.^52^ There are a few remarks about these thermoplastic materials, which differentiate their application scenarios. PS is a suitable material for cell culture. Also, it can be adapted for quick multilayer fabrication with thermal bonding. PC is widely used for molecular diagnostic applications due to its transparency and high glass transition temperature (∼145 °C), which means it can satisfy the temperature requirement during nucleic acid amplification. Multilayering of PC is possible while the bonding condition needs consideration for a tight seal and undistorted channels. PMMA, also known as Plexiglas and Acrylic, is a cheap and versatile material that can be utilized specifically as disposable microfluidic chips. COC and COP are amorphous polymers with low water absorption and excellent chemical resistance based on different polymerization processes. They also have a high optical transmissivity in the ultraviolet (UV) range, which enables the use of photoinitiators, such as benzophenone for bio-interface applications.^53^ Except for the chip itself, plastic materials, such as PC-ABS and PP, could be used to fabricate the cartridge housing and chip-to-world connections. Their lower prices can help reduce the overall chip cost in the scale-up stage.

**Paper:** Paper-based microfluidic devices employ capillary force as the liquid driven force to provide a rapid, disposable, and affordable method for medical diagnosis and biochemical analysis. Detection of analytes in paper microfluidics includes colorimetric detection, electrochemical detection, fluorescent detection, chemiluminescent detection, and so on.^54^ Among them, colorimetric detection is the most common method, which is typically related to enzymatic or chemical color-change reactions. Paper-based microfluidic devices are not compatible with applications that require optical transparency or complex sample manipulation; however, they still play an important role in the POCT market for applications with relatively low sensitivity and selectivity.^3^

#### 3. Fabrication techniques in prototyping

##### a. Wet/dry etching (silicon/glass)

Micromachining of silicon and glass covers patterning process (UV photolithography and electron beam lithography) and etching process (wet etching and dry etching). During the patterning process, the photoresist is first spin-coated on the substrate, followed by exposure to UV light or electron beam. UV photolithography can achieve the minimum feature size of around 0.5 *μ*m in seconds to minutes, while e-beam lithography needs hours to reach a much higher resolution around 6 nm. After the photoresist is selectively dissolved in a developer solution, etching is carried out. Reactive ion etching (RIE) is a frequently utilized method for dry etching. RIE employs a chemically reactive plasma containing charged ions to bombard the material surface and react with it to create etching cavities with higher aspect ratios. For wet etching, the unprotected surface of the substrate will be dissolved when immersed in a liquid etchant solution. Wet etching is usually an isotropic process, which is recommended for glass. While deep RIE is a highly anisotropic process, it is more versatile and reliable for silicon etching. Suitable etchants for glass and silicon are listed in detail by Iliescu *et al.*, together with the summary of bonding methods that are used for joining these materials.^56^

##### b. Soft lithography (PDMS)

Soft lithography refers to the fabrication of elastomeric mold usually in PDMS and a collection of techniques that use PDMS as an elastomeric stamp for the printing, embossing or molding of microstructures or nanostructures. These patterning techniques generally encompass microcontact printing (*μ*CP), replica molding (REM), microtransfer molding (*μ*TM), micromolding in capillaries (MIMIC), and solvent-assisted micromolding (SAMIM).^57^ Here, the PDMS structure can work independently as a microfluidic device, which is commonly carried out in laboratories as a quick method for concept validation. Fabrication of PDMS can be simplified as three steps: master mold fabrication, PDMS casting, and curing by baking. Standard UV photolithography is used for making microsized master molds with complementary patterns and e-beam lithography is mainly used for nano-sized structures. After master mold fabrication, the PDMS precursor is poured or spin-coated on the master mold, followed by a degassing process in a vacuum. When there are no bubbles inside, a PDMS prepolymer can be cured by baking at 70 °C for 6 h, after which it can be peeled off with a replica pattern on the surface. Sylgard 184 from Dow Corning is one widely used elastomer for PDMS fabrication.

##### c. Micromilling (plastic)

Micromilling represents a material removing technique with the assistance of a high-precision computer numerical controlled (CNC) machine. This mechanical method is suitable for materials including metals, polymers, composites, and ceramics.^58^ Due to its simplicity and short turnaround time, together with the relatively low cost of the CNC machine, micromilling is an attractive method for straightforward fabrication of polymeric chips in the prototyping process.

##### d. Laser-based manufacturing (plastic, glass, paper)

Laser ablation takes the advantage of an intense continuous or pulsed laser beam to remove irradiated parts from solid materials. Heat generated around the beam spot is absorbed by the material, resulting in evaporation, sublimation, or conversion to plasma if the laser flux is high enough. Similar to the micromilling process, laser ablation is more accessible compared with the MEMS techniques with cleanroom facilities. The minimum feature size can reach below 1 *μ*m, which is good enough for most microfluidics-based applications.^59^ The potential drawbacks include limited throughput, surface non-uniformity, and small variations between different slots. But still, laser ablation is widely applied for research projects as a one-step fabrication method that is also cost-effective and environmentally friendly. Laser ablation is mainly used for non-contact cutting of the device edge or thin-film structures like plastics, papers, or adhesive tapes. These thin layers can be removed individually and then assembled as laminate microfluidic devices. The simplest one consists of an interface layer, a flow layer, and a bottom layer. Laser cutting offers a straightforward option for thin-film manufacturing with low cost, high resolution, and short working time. A knife plotter, if a lower resolution is required, can replace the laser cutter to create the geometry of laminate microfluidic devices. This method is also known as xurography.^60^ PDMS and COC films can be directly structured using xurography.

##### e. 3D printing (plastic)

When it comes to a geometrically complex design, conventional methodologies mentioned above may face difficulties while 3D printing can offer a smart option for accurate layer-by-layer manufacturing of the material. 3D printing has gained much attention recently, especially for the early-stage development of microfluidic devices due to cost-effectiveness, capability for complex structures, and short fabrication time. A variety of techniques have been exploited based on 3D printing, such as fused deposition modeling (FDM), stereolithography, digital light processing (DLP), Polyjet 3D printing,^61^ and two-photon polymerization (2PP).

FDM extrudes melted thermoplastic filament from a heated nozzle and form solidification by cooling.^62^ The nozzle is removable so that the melted material is deposited in a layer. Stereolithography and DLP both belong to vat photopolymerization, which employs a UV light source for the curing of photopolymer resins in the liquid.^63,64^ The difference is stereolithography uses a UV light beam and hardens the material point-by-point, following the movement of the beam spot. For DLP, the light is emitted from a projector and the curing process is finished one layer at a time. Polyjet 3D printing combined ink-jet technology with UV curable materials to produce smooth and detailed prototypes, also one layer at a time.^65^ Two-photon polymerization takes advantage of two-photon absorption and is the most precise 3D printing process that enables the fabrication of 3D objects with spatial resolution below 100 nm.^66^ Which 3D printing technology to choose depends on material selection, the resolution required and structure complexity. Not only can it work for the manufacturing of microfluidic chips or the main body of cartridges, but 3D printing can also produce fixtures or jigs in a short time. It is an ideal additive manufacturing technique with much allowance for design freedom, and, predicatively, it will accelerate the development of innovative microfluidic devices in the prototyping stage.

#### 4. Back-end process in prototyping

To enrich the functions inside a microfluidic chip, the back-end process is becoming an indispensable part of the manufacturing supply chain. It covers reagent storage, surface treatment, chip bonding, and other steps depending on the required level of integration and automation. Techniques for each step shown here are much similar to those in the scale-up fabrication process. The main difference is that for the prototyping stage, the back-end process is mostly finished by hand, while for mass fabrication, a robust and streamlined automatic system is usually developed to increase manufacturing efficiency. Detailed information on various back-end processes will be introduced in the scale-up fabrication stage later.

### B. Scale-up production stage (high volume: >10 000 pieces)

As for scale-up production, the purpose is to translate the proof of concept into repeatable low-cost production in high quality and large quantity for clinical testing and customer use. The development of microfluidic devices for high-volume fabrication, as illustrated in Fig. 2, generally covers the following steps: mold fabrication, replication, back-end process, and review process. We will emphasis on the scale-up fabrication of thermoplastic chips when addressing techniques for mold fabrication and replication. As for the back-end process, materials including silicon, glass, and PDMS will also be covered. It should be noticed that some of the technologies for scale-up production, such as injection molding, have also been used for prototyping and small quantity production in industry by using a single or dual cavity prototyping mold. The processes that we mentioned were selected based on our industrial practice for scale-up production of plastic microfluidic chips.

#### 1. Mold fabrication

Thermoplastic chips generated from a mold will guarantee the consistency of products at high efficiency and low cost per piece. Mold tool insert for microfluidic chip molding is a metallic part, usually made from tool steel or nickel, containing inverted micro/nanochannel structures. It is assembled with a mold frame to compose an injection mold for scale-up production of microfluidic chips. Since the production volume can range from several hundred parts to tens of thousands and even millions of parts, requirements for mold tool inserts are quite rigorous.

Here, we summarize the requirements for mold tool inserts in mass production:

(1) precise patterning with required feature size and aspect ratio,
(2) sufficient strength and durability,
(3) good demolding capability with a feasible draft angle,
(4) appropriate surface finish, and
(5) good cost/performance ratio.

The commonly used materials for a microstructured mold tool are tool steel and nickel. A tool steel microstructured mold is generally made by micromachining, while a nickel mold is fabricated by the microelectroforming process.

##### a. Micromachining

The currently available methods for mold fabrication are diverse. Micromilling and laser ablation, which are mentioned in the prototyping process for direct fabrication of polymer chips, are also prevalent for mold tool insert fabrication. Micromilling is easily accessible and relatively cheap for 3D metallic fabrication with materials of brass, aluminum, and stainless steel. However, the drawbacks like machining burrs, a low aspect ratio of microfeatures and relatively high roughness need to be noticed. Ultra-precision micromilling using diamond tools/cubic boron nitride (CBN) tools could achieve a good surface finish. However, the overall cost including milling tools and machining time can be high. Microelectrodischarge machining (*μ*EDM) is a promising micromold fabrication process for tool steel. It is an electro-thermal machining process that removes the material from workpiece by means of the electrical discharges between tool electrodes and the work piece electrode.^67^ Compared to micromilling, *μ*EDM could achieve a better surface finish with machining burrs and tool tracks. However, a *μ*EDM machine is not easy to access and the machining process is also slow. For both micromilling and micro-EDM, the feature size is generally limited to be over 100 *μ*m based on the industrial practice, although academic research has achieved a smaller size, which is still not practically realized in the industry.

##### b. LIGA

LIGA technology is another important metallic mold fabrication process. LIGA is a German acronym for LIthographie, Galvanoformung and Abformung, which corresponds to lithography, electroplating, and molding. Depending on the light source for lithography, LIGA has two main groups. One is using x rays for structures with high aspect ratio; the other is using UV light to create features with a relatively lower aspect ratio. In LIGA process, the substrate is a flat surface with electrically conductive material. A silicon wafer with a conductive layer pre-deposited through evaporation or sputtering is also a proper choice. By lithography, a resist structure is patterned on the substrate after development. For electroforming, the substrate is then placed into a nickel sulfamate electrolyte for electrodeposition of a thick layer from 0.5 to 5 mm thickness. After the etching of silicon substrate and photoresist, the electrodeposit is left as a mold with subsequent cutting. Nickel tool, compared to stainless steel tool, is soft with hardness at around 200 HV. Tool steel could have a higher hardness ∼600 HV.^68^ Currently, novel strategies of nickel alloy and nickel composite have been developed for tool fabrication by our team. At the moment, we do not have the direct data of microstructured tool life. Based on our industrial practice, pure nickel mold can be used for more than 10 000 molding cycles without problems. Nickel mold is generally used for microfluidic channel with a size smaller than 100 *μ*m and surface roughness below 50 nm. A comparison between micromilling, micro-EDM, and UV-LIGA is given in Table II for a reference in tooling method selection.

**TABLE II.** Comparison between different tooling methods.^59,69^

Technology	Minimum feature size	Aspect ratio	Surface roughness (Ra)	Feature tolerance	Mold materials
Micromilling^a^	50 *μ*m for sunk features	1.5 (features in the range between 50 and 100 *μ*m)	0.1–1 *μ*m	5 *μ*m	Stainless steel, brass, aluminum
*μ*EDM^a^	5 *μ*m	<20	100 nm	3 *μ*m	Stainless steel, titanium
UV-LIGA	50 nm–500 *μ*m	<20	15 nm	5% channel size	Nickel, copper, nickel alloy

^a^ Note: Industrial practice of tool fabrication indicates feature more than 100 *μ*m is commonly achievable for micromilling and micro-EDM.

#### 2. Microreplication process

The predominant methods for plastic chip replication are microinjection molding and microhot embossing,^70^ which will be introduced below.

##### a. Microinjection molding

For injection molding, the thermoplastic material is melted by heating and shearing in plasticization, followed by injection at high pressure into the cavity between two molds and by packing to compensate shrinkage during material solidification. When the material is cooled and solidified, the injection molded part can be ejected from the mold. Injection molding is a well-established technique for mass production, especially for disposable products in medical applications, due to its easy automation, high repeatability, and low cost per unit. The injection molding cycle is usually less than a minute with a high production capability. It is the only technology to produce complex 3D geometry of the microfluidic cartridge.

The high tooling cost is one of the drawbacks of injection molding. Dependent on the production volume, mold design can be a single cavity mold or multicavity mold. Single or two-cavity mold is generally used for prototyping and low quantity production. It is also noticed that the high aspect ratio in mold geometry will increase the probability of insufficient filling. New technologies, such as variotherm assisted injection molding, heat transfer retardation technique, vacuum venting, and non-adhesive surface coatings, have been developed to combat this problem.^71–73^ Other novel technologies, such as in-mold microcompression, have been used to push a hot polymer melt into the micro/nanomold cavities to increase the replication fidelity of microscale and nanoscale features, which extends the microinjection molding capability for higher aspect ratio and narrow entrance features.^74^

##### b. Microhot embossing

Hot embossing is a process commonly used for thermoplastic structures, typically in the form of thin films or plates, to be patterned with a stamp mold. During embossing, film and molds are heated to or above glass transition temperature of the polymer. Under a high contact pressure, patterns from molds are transferred into the softened polymer surface and solidified by cooling. Compared with microinjection molding, hot embossing has a higher replication accuracy and lower cost on mold tooling. Moreover, it is capable for features with a higher aspect ratio. However, it cannot fabricate microfluidic chips with 3D structures, such as chip-to-world connectors. Microhot embossing is only used for small quantity prototyping and the cycle time can be 10 min.

To increase the production efficiency, a high throughput roll-to-roll (R2R) hot embossing system is generated for continuous duplication of polymer thin films with microfluidic patterns,^75^ where roller-shaped molds with inverted micropatterns can be used. However, R2R hot embossing can be only used for thin-film materials. This technology is still under development. The subsequent bonding of thin-film microfluidic chips is also challenging. Some features of these three replication methods are listed in Table III for a better comparison.

**TABLE III.** Comparison between injection molding, hot embossing, and R2R imprinting.

		Injection molding	Hot embossing	R2R imprinting
Mold cost	High		Low	Low
Unit cost	Low		Low	Low
Cycle time	∼30 s		tens of minutes	seconds
Complexity of part geometry	3D		2.5D	limited to thin films
Aspect ratio	Low		High	Low
Suitable for low quantity	Dependent on quantity		Yes	Yes
Commonly used for	Low-cost scale-up production and 3D complex geometry		High-precision and high-quality microstructures	Large area nano/micropatterns

It is worthwhile to mention that multilayer lamination can be also a scalable process for a low-cost microfluidic chip fabrication, such as LumiraDx test strips. The future development trend of the microfluidic chips could be either based on high throughput film-based chips or complex 3D chips from injection molding. The current industrial technology used in other fields, such as screen printing, will be borrowed to scale-up production of microfluidic chips.

#### 3. Back-end process in the scale-up fabrication

Back-end process, including reagent storage, surface treatment, bonding, and integration, are essential steps for microfluidic chip scale-up. Compared to mature replication technologies, such as injection molding, a back-end process would need to take R&D efforts. The current back-end process accounts for 70% of the total fabrication cost due to immature automation and limited capability for batch production. Here, we discuss the major back-end processes.

##### a. Reagent storage

To reach a fully automated system, especially for the short time requirement in POCT applications, reagents and/or buffers must be stored inside the cartridge before sample injection. There are plenty of reagent types, including enzymes, primers, antibodies, substrates, aptamers, and magnetic beads.^11^ Buffers can be utilized for sample lysis, binding, washing, dilution, and elution. Design of the reagent storage reservoir is related to parameters like reagent type, storage condition, handling/dispensing method, releasing time and sequence, etc. The filling trajectory of a liquid in the reagent storage chamber is also a concern because reagents are possible to dissolve so fast that they accumulate at the liquid front, causing a uniformity of mixture concentration.

Storage of dry reagents includes dispensing and successive drying of liquid reagents within the cartridge chamber. In other cases, liquid reagents are lyophilized into pellets and handled into the chamber afterward. Most integrated cartridges prefer the storage of solid reagents, including lyophilized reagents, due to the desired shelf time and high resistance to temperature change.

Liquid reagents are more stored inside blister pouches^76^ (Abbott laboratories), stick packs,^77^ and tanks with wax valves^78^ (GenePOC™). Researchers also explored many other methods for the integration of reagents and active releasing systems such as glass ampoules,^79^ capillary,^80^ and PDMS sponge.^81^ Hitzbleck and Delamarche gave a detailed review on integrating and releasing reagents in dry and liquid forms in microfluidics.^10^

During cartridge manufacturing and assembly process, the following aspects should be considered in the aspect from both liquid and dry formats:

(1) Cartridge material: to satisfy chemical and/or biological compatibility.
(2) Assembly sequence: to protect reagent in filling and long-term storage (filling of reagents can be performed in the last steps of the manufacturing process chain).
(3) Opening mechanism: to protect reagent in the leasing step.
(4) Temperature control: to reduce reagent disruption and evaporation.

##### b. Surface treatment

Surface treatment is significant for multiple requirements in microfluidic devices, such as facilitating cell adhesion,^82^ improving surface wettability,^83^ and increasing bonding strength between layers.^84^ Take PDMS as an instance. Its hydrophobic nature makes it difficult to introduce aqueous solutions into the PDMS-based microchannels.^85^ Moreover, its non-specific adsorption of proteins and other molecules limits its broader use.^83^ To solve these problems, various surface treatment methods for PDMS microfluidic devices have been employed including plasma oxidation, ultraviolet (UV) irradiation, chemical vapor deposition (CVD), solgel coating, and silanization.^85^ On the other hand, the feature of hydrophobicity is preferred for the stable generation and transfer of water-in-oil droplets.^86^ For industry usage, methods for surface treatment should be rapid, inexpensive, and suitable for batch production.

##### c. Assembly/bonding

After the fabrication of each component inside a cartridge, a critical step is to assemble them into a whole part. Bonding process is of great importance for the combination of two interfaces to produce an enclosed fluid path. Bonding strength and interface properties need full consideration during the fabrication process to make sure (1) there is no excessive deformation of microchannels, (2) the interface is chemical or biological compatible, and (3) no leakage occurs.

Depending on whether an adhesive layer is needed or not, bonding techniques for PDMS and glass chips can be categorized as direct and indirect types. Indirect bonding requires an intermediate layer, such as double-sided tape or glue, to seal two substrates. Others materials like SU-8 resist^87^ or a partial-cured thin film of PDMS^88^ can also be employed as a sacrificing adhesive layer. As for direct bonding, the substrate material itself comprises the adhesive without any additional material, such as plasma activation, welding (laser/ultrasonic/microwave), thermal fusion bonding, solvent bonding, and anodic bonding. Many of the techniques involved also have a temperature requirement during the bonding process. Under some circumstances, other steps like UV irradiation or surface modification are involved to ensure the bonding efficiency.^89^ For example, oxygen plasma treatment followed by surface modification with 1% (3-aminopropyl)triethoxysilane (APTES) and (3-glycidoxypropyl)trimethoxysilane (GPTMS) is displayed to realize bonding between PDMS substrates at room temperature.^90^

For thermoplastic microfluidic chips, the commercial mature process includes ultrasonic bonding, laser bonding, thermal diffusion bonding, and gluing. Ultrasonic bonding can be a stable and fast bonding process. However, it requires energy directors, e.g., wedge-shaped extrusion around the microchannel of plastic chips for reliable bonding, and the relative melting zone can be large, which might block and deform the tiny channels. Ultrasonic bonding can be used in the integration of foils, connectors, and large parts of the microfluidic cartridge. Laser bonding is another industrial bonding technology for plastic materials. It is fast and well established. However, it requires at least one colored plastic substrate for laser energy absorption. It may not be suitable for small channel bonding, as it could induce local channel deformation during melting and compression. Thermal diffusion bonding is well used in the laboratory for plastic chip bonding, as it requires a less expensive hot press. Assisted with solvent vapor deposition, thermal bonding can achieve very high bonding strength up to several MPa. However, it still has problems of channel distortion and requires much effort for optimization. Compared to ultrasonic bonding and laser welding, the cycle time of thermal diffusion bonding can be longer, up to several minutes. Gluing is well used for structural bonding, and it is mainly used in the microfluidic industry for the integration of multimaterials, such as bonding of glass to printed circuit board (PCB) and PCB to thermoplastic chips. It is important to mention that proper cleaning of single-layer microfluidic chip and plastic film is of great importance for successful bonding. Additionally, a cleanroom environment is preferred for bonding microfluidic chips. Bonding methods for chips with various materials are concluded in Table IV.

**TABLE IV.** Commonly used bonding techniques between various materials.

	Silicon	Glass	PDMS	Thermoplastic
Silicon	Fusion bonding			
Glass	Fusion bonding Anodic bonding Adhesive bonding Laser welding	Fusion bonding Plasma activation		
PDMS	Adhesive bonding	Oxygen plasma	Oxygen plasma	
Thermoplastic	Adhesive bonding	Thermal bonding Adhesive bonding	Adhesive bonding	Ultrasonic welding Thermal bonding Laser welding Adhesive bonding

##### d. Other processes

For a complete fabrication workflow of a microfluidic cartridge, some other processes may need further attention, as follows:

(1) Metallization of electrodes on plastic chip for a broad spectrum of applications including droplet manipulation using electro-wetting on dielectric (EWOD) in companies like Advanced Liquid Logic^91^ (acquired by Illumina Inc. in 2013) and Miroculus,^91,92^ and impedance-based diagnostics.^93^
(2) Flip-chip bonding for integrating complementary metal–oxide–semiconductor (CMOS) sensors with microfluidics.^94^
(3) Other mechanical processes like port drilling and chip dicing.
(4) Packaging of the final product.

It would be a sophisticated process to go through from the very beginning to the final step in the fabrication workflow. By dividing the process into multiple steps, together with a sketch of the most common techniques involved, we hope to provide a better conception here when it comes to cartridge design or fabrication method selection.

## V. STANDARDIZATION

Voices from many corners for the standardization of microfluidic-based devices have been heard for years. The core demand is to establish a set of standards to accelerate the commercialization of microfluidic-based medical devices,^20^ which cover topics about interfacing, fluidics control, testing protocol, and modularity. These standards are offering guidelines for (1) dimension and performance control between microfluidic connections, (2) precise measurement of flow rate, (3) reliable tests on various components or device features, and (4) modular approach to integrate microfluidic, electric, and optical functionalities.^20^ To reach the goal, interested parties including regulatory stakeholders, academic researchers, and industry representatives participate in the standardization activities. SEMI (Semiconductor Equipment and Materials International) and ISO (International Organization for Standardization) have published some standards to address interconnections in microfluidic systems, as listed in Table V. Some of them are still under development.

**TABLE V.** Standards developed by SEMI and ISO.

Association	Standards of interest	Status
SEMI	SEMI MS6-0308 Guide for Design and Materials for Interfacing Microfluidic Systems	Published
	SEMI MS7-0708 Specification for Microfluidic Interfaces to Electronic Device Packages	Published
	SEMI MS9-0611 Specification for High Density Permanent Connections Between Microfluidic Devices	Published
	SEMI MS11 Specification for Microfluidic Port and Pitch Dimensions	Published
ISO	IWA 23:2016 Interoperability of microfluidic devices—Guidelines for pitch spacing dimensions and initial device classification	Published
	ISO/DIS 22916 Microfluidic devices—Interoperability requirements for dimensions, connections, and initial device classification	Under development
	ISO/AWI TS 6417 Microfluidic pumps—Symbols and performance communication	Under development

Standardization of microfluidics is of great help for building up a regulated and harmonized supply chain in industry. From the manufacturing aspect, it cuts off the redundant work in the device design process, like deciding the dimension of inlet/outlet ports; thus, more effort could be devoted to other core functions. Fabrication of standard interconnectors or modular structures will cost less due to the largely increased quantity. Moreover, standards for integration and modularity will significantly reduce the development time of products, especially in prototypes. It is never an easy task to set a standardization system in any industry, not to mention microfluidics with such a wide variety of potential applications and fabrication processes. However, for long-term development, more participants in the microfluidic community should be united to speed up the standardization process.

## VI. SUGGESTIONS FOR CHALLENGES

Before the birth of one commercial cartridge from the mass fabrication process, manufacturers have to face all kinds of challenges from material selection to integration solutions. They are responsible for product quality control, especially of medical devices, and for reducing cost and expanding margin as well. To thrive, not only survive, in the competition of this emerging market, a well-organized product plan based on a thorough market analysis is always necessary in the first place. Ulrich *et al.* divided the development process of a general product plan into five steps: opportunity identification, evaluation and prioritization of opportunities, time and resource allocation, completion of pre-project planning, and reflection on the results and the process.^42^ All these steps can be applied to the product development of microfluidic devices.

Once the project plan is defined, subsequent manufacturing would become a key problem. To conquer the challenges mentioned in Sec. II, we concluded four suggestions for a better translation of microfluidic cartridges in different development phases, on the basis of our experience in MiNAN technologies^95^ and collaboration with other stakeholders:

(1) Generate a good design for the ease of manufacturing.

As you can see from Fig. 2, the development of microfluidic devices has a long process chain, involving many different micromanufacturing processes like micromold tools, microreplication, microscale integration, etc. Except for the difficulties in single process optimization, process iteration is always necessary to have qualified products. A broad design for manufacturing analysis from the experience of conventional products cannot be applied to microfluidic chip scale-up directly, particularly when the microchannel dimensions are smaller than 100 *μ*m. This could be the most challenging part for microfluidic chip scale-up production and requires a good amount of attention. A good design can save much effort. For example, channels with rectangular or square cross sections are preferred due to the high difficulty in obtaining rounded cross sections when making the mold using etching, LIGA, or micromilling; a lower aspect ratio reduces the risk of structure break/distortion during demolding of the injection molding process; a positive draft angle of the microchannel helps well in the separation of tooling and chip part; channels and ports need a safe distance from the chip edge to avoid sporadic leakage for bonding; be careful with the arrangement of channels against the gate and runner design of the mold, where channel replication uniformity and tolerance are important. Checking the design tips of channels, chambers, and ports in microfluidic chips in the design and prototyping stage by considering the potential challenges of scale-up will eventually decrease turnaround time and save cost. Importantly, use larger channels if it is not necessary to be that small. It will save much effort from tooling, replication, and integration. Overall, a good design can save much effort in the subsequent scale-up and many challenges can be well avoided.

(2) Involve manufacturing professionals in the early stage of development.

Manufacturing feasibility should be a concern from the very beginning of the product development life cycle. Experts with professional knowledge will give suggestions to streamline the manufacturing process with high feasibility and efficiency. This can effectively reduce failure cases toward scale-up production, particularly for those generated from academic projects, which are caused by manufacturing problems like wrong materials or immature techniques. As many micromanufacturing processes have been involved in the microfluidic chip development process, not every manufacturer could provide such professional advice. Some professional micromanufacturing companies have accumulated enough knowledge in their manufacturing process, but these experiences can be confidential and cannot be shared. An additional obstacle is that large companies may not be interested in working with scientists from research organizations, as their projects could be mostly in the early stage and cannot reach mass production in a short time. Usually, the research project from the laboratory is constrained by funding but requires high-level expertise in micromanufacturing, which is not of good interest to large manufacturing companies. At the beginning of a project, involving a professional who has experience in micromanufacturing and microfluidic cartridge scale-up can be important. In Sec. IV, we selected important scale-up manufacturing processes for scale-up production of plastic microfluidic devices, which can help researchers to primarily select the required processes for engagement with manufacturing professionals. This can save much effort in the iteration of development if people really want to convert their prototyping to a commercial product.

(3) Use commercial mass production materials if possible.

As the materials used in laboratory can be different from that in the scale-up production, a full understanding of material differences in terms of surface properties (e.g., wettability), gas permeability, mechanical properties, and manufacturability (e.g., replication fidelity, surface treatment, bonding method) can be important. Such knowledge is required to reduce the potential cost of redevelopment. A comprehensive understanding of assays running on the chip and their requirements in terms of materials is important to decide the proper material that can be used for prototyping. As PDMS has been well used in the lab, while the industry scale-up prefers thermoplastics,^96,97^ understanding of the differences between PDMS and thermoplastics is important in terms of chip design and development. Our view is that, if possible, thermoplastics should be used, if the objective is the subsequent translation. Of course, cost and accessibility can be an issue.

(4) Use standards and join in the standardization work if possible.

Although there is a long way to go for standardization in microfluidics, it is promising that standardization will boost the adoption of microfluidics by the market as it has done and is still doing to electronics. Keep an eye on the update of standards that are published or still under development and take them as a reference for chip design or testing. It will bring benefits like greater flexibility, reduced development time and consistent quality when using standard components like interconnectors. The standardization process is promoted voluntarily nowadays. If more efforts are involved, the system would be established and improved in a shorter time. Except for the standards of microfluidics, the microfluidic cartridge is broadly categorized as medical devices. For cartridge development, using existing macroworld connectors and making the system compatible with existing laboratory instruments are important to improve the adoption of the system. Importantly, start with the chip development and then the instrument development, as instrument is highly dependent on the chip design and external connection.

## VII. CONCLUSIONS AND OUTLOOK

The expansion of the microfluidic device market attracts more researchers to devote themselves to this emerging field. No matter they join a big company or build a start-up, manufacturing is always an important issue during the development of a product. If industries do not have in-house manufacturing technologies, outsourcing will be a good choice. In such cases, their manufacturing engineers will join the core project team in the early stage. For those who are handling the transition on their own but with limited experience, a picture of the essential processes ahead should be much helpful.

In this Perspective, we attempt to give a brief but clear introduction of the workflow in two different development periods (prototyping and scale-up fabrication period) with focus on the manufacturing techniques involved. Here, the prototyping period is referred to as academic research or industrial research at the early stage while the scale-up production is more related to a larger-quantity production using industrial mass production technologies to satisfy the requirements of microfluidic chips for commercial usage. Given the development process of microfluidic devices discussed above, we want to share our understanding on the challenges and offer some suggestions correspondingly based on our experience. We hope a manufacturing mindset is built and more attention will be paid to a good design and a robust protocol. Also, collaborations between various disciplines are always encouraged to establish the standardization system and accelerate the commercialization of microfluidics.

## Data Availability

The data that support the findings of this study are available within the article.
